# Lipid droplet-associated proteins: Roles in cardiovascular diseases

**DOI:** 10.1016/j.jlr.2026.101010

**Published:** 2026-02-25

**Authors:** Zhongyang Yu, Meng Zhao, Hongyue Xu, Xiaoxing Jin, Ji Sun

**Affiliations:** 1 State Key Laboratory of Cardiovascular Diseases, Shanghai East Hospital, School of Medicine, Tongji University, Shanghai, China; 2 Shanghai Frontiers Center of Nanocatalytic Medicine, Tongji University, Shanghai, China; 3 Department of Pathology and Pathophysiology, School of Medicine, Tongji University, Shanghai, China; 4 Clinical Center for Heart Disease Research, Tongji University, Shanghai, China; 5 Shanghai Arrhythmia Research Center, Shanghai East Hospital, School of Medicine, Tongji University, Shanghai, China; 6 Department of Cardiology, Shanghai East Hospital, School of Medicine, Tongji University, Shanghai, China; 7 State Key Laboratory for Diagnosis and Treatment of Severe Zoonotic Infectious Diseases, Key Laboratory for Zoonosis Research, Ministry of Education, Institute of Zoonosis, College of Veterinary Medicine, Jilin University, Changchun, China; 8 Department of Cardiology, The First Affiliated Hospital, Sun Yat-sen University, Guangzhou, Guangdong, P.R. China; 9 Department of Cardiovascular Medicine, Fifth Affiliated Hospital of Sun Yat-sen University, Zhu Hai, P.R. China; 10 Department of Cardiology of Central China Fuwai Hospital, Central China Fuwai Hospital of Zhengzhou University, Zhengzhou Henan, P.R. China

**Keywords:** cardiovascular diseases, lipid droplets, lipid droplets associated proteins

## Abstract

Cardiovascular diseases (CVDs) are among the leading causes of morbidity and mortality worldwide. In the cardiovascular system, lipids serve as a primary energy source, and dysregulated lipid metabolism has been observed in many CVDs. Lipid droplets (LDs) are organelles that store lipids, including triglycerides and cholesterol. The biogenesis and lipolysis of LDs broadly influence lipid metabolism in cells in the cardiovascular system and contribute to CVDs. LDs homeostasis is modulated by lipid droplet-associated proteins (LDAPs), such as PLINs, CIDEs, BSCL2, ABHD5, and Rab18. These proteins have also been reported to be involved in various CVDs. To our knowledge, this is the first review to systematically elucidate the associations between LDAPs and CVDs. Here, we summarize the roles of LDAPs in CVDs and discuss them in detail.

Cardiovascular disorders (CVDs) represent the primary contributors to global mortality and morbidity rates. Owing to the harmful effects of CVDs, researchers have directed efforts toward elucidating their pathogenic mechanisms across scales—from macroscopic to microscopic—with the goal of identifying new therapeutic approaches. Recent studies have identified metabolic disturbances in CVDs, revealing that particular metabolic pathways and their associated metabolites are critically involved in disease progression. The cardiovascular system relies predominantly on lipids as an energy substrate, and proper lipid metabolism is crucial for preserving normal physiological activity. Disruptions in lipid metabolism, including compromised fatty acid β-oxidation, contribute to the onset and development of various CVDs. Lipid droplets (LDs), organelles derived from the smooth endoplasmic reticulum (ER), function to store neutral lipids within cells. Cellular lipid metabolic status is regulated by the abundance and dimensions of LDs, a mechanism under the control of lipid droplet-associated proteins (LDAPs).

In this review, we address the metabolic changes observed in CVDs and the role of LDs in modulating lipid metabolism. Next, we examine the LDAPs implicated in the formation and degradation of LDs. Lastly, we provide a synthesis of the involvement of LDAPs in CVDs and the emerging and prospective therapeutic opportunities associated with targeting these proteins.

## Lipid Metabolism in CVDs

Under normal physiological states, the myocardium exhibits high energy demands, relying heavily on lipid metabolic processes for ATP generation. Consequently, pathological disturbances in lipid homeostasis frequently compromise cardiac function and predispose the system to CVDs. This section outlines the critical role of lipid metabolism across various CVD pathologies.

### Atherosclerosis

Atherosclerosis is a common group of vascular diseases characterized by the accumulation of lipids and chronic inflammation in the large arteries, ultimately contributing to the development of stroke and coronary artery disorders including angina and myocardial infarction (1). In the progression of atherosclerosis, low-density lipoproteins (LDLs) and triglyceride-rich lipoproteins are deposited in the subendothelial space. Subsequently, monocytes circulating in the plasma bind to adhesion molecules expressed on the surface of endothelial cells, transmigrate into the intima, and differentiate into macrophages. Then these macrophages internalize the lipoproteins and transform into foam cells that are heavily loaded with cholesterol esters. As a result, a complex aggregate composed of lipids, necrotic debris, and various cell types—including smooth muscle cells, endothelial cells, and immune cells—accumulates to form an atherosclerotic plaque between the tunica intima and tunica media. When vulnerable plaques rupture, the ensuing thrombotic occlusion impedes arterial blood flow, leading to acute myocardial infarction and ischemic stroke (2).

Although atherosclerosis can be triggered by numerous factors, hyperlipidemia represents a major predisposing condition. The initiation of atherosclerosis is marked by endothelial dysfunction, a pathological alteration that modifies arterial susceptibility to hyperlipidemia. As a consequence of disrupted endothelial barrier function, elevated plasma levels of LDLs accumulate beneath the intimal layer, thereby promoting plaque formation (3). Thus, understanding the mechanisms underlying endothelial dysfunction is essential for the prevention and treatment of atherosclerosis. Nitric oxide (NO) has been recognized as a central mediator in endothelial dysfunction. The scavenging of NO by oxidative enzyme systems—such as NADPH oxidase, cyclooxygenases, and myeloperoxidases—results in a marked increase in superoxide (O_2_^-^), which contributes to endothelial dysfunction (4). With respect to NO synthesis in endothelial cells, studies have demonstrated that free fatty acid (FFA)-associated lipid metabolism can modulate this process through the AMPK/PI3K/Akt/eNOS pathway (5) and calcium-dependent signaling pathways (6). When these signaling pathways are impaired, endothelial NO production is reduced, leading to endothelial dysfunction. Furthermore, FFAs can suppress NO synthesis by activating the NF-κB (7) and NLRP3 (8) inflammatory pathways. Considering the critical role of FFAs in endothelial dysfunction, limiting FFA uptake into endothelial cells may help preserve cellular function (9). Additionally, diminished fatty acid oxidation within endothelial cells can contribute to endothelial dysfunction. Endothelial cell–specific deletion of the ATGL gene promotes lipid accumulation, increases arterial stiffness, and accelerates the onset of atherosclerosis (10, 11). In conclusion, FFA-associated lipid metabolism plays a role in regulating NO production in endothelial cells and maintaining endothelial barrier integrity.

**Fig. 1:**
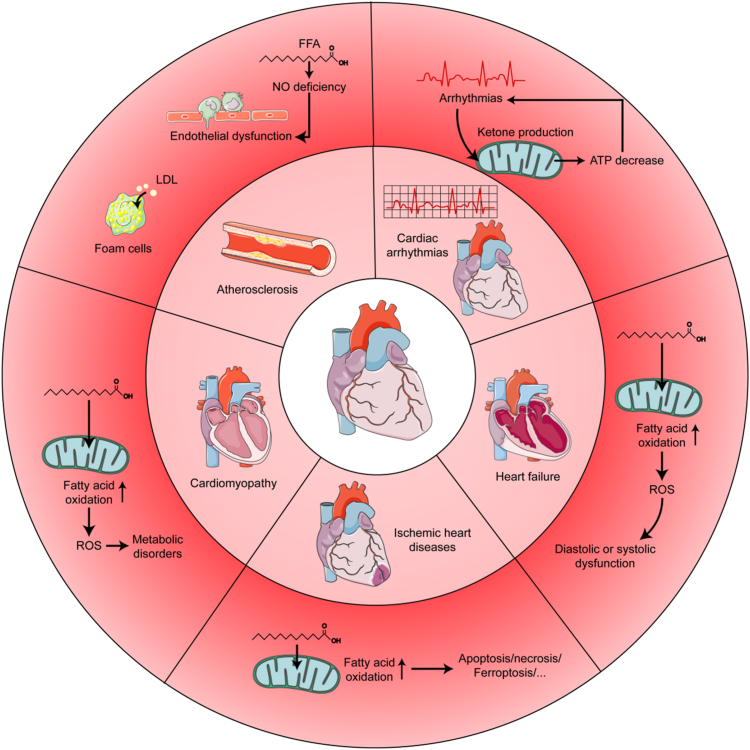
A representative diagram of lipid metabolism changes in diverse CVDs. Lipid metabolism plays different roles in CVDs. In atherosclerosis, free fatty acid (FFA) in plasma can induce NO deficiency in the endothelium and thereby contribute to endothelial dysfunction. In the meanwhile, macrophages can absorb low-density lipoprotein (LDL) and then transform into foam cells, which promotes the progression of atherosclerosis. For cardiomyopathy, excessive fatty acid oxidation in cardiomyocytes promotes the production of ROS in mitochondria. Similarly, increased myocardial fatty acid oxidation can lead to cell death such as apoptosis, necrosis, and ferroptosis during ischemic heart diseases. Excessive fatty acid oxidation can also lead to heart failure via reactive oxygen species (ROS) related pathways. During cardiac arrhythmias like atrial fibrillation (AF), consistent arrhythmia is criminal for ketone production in mitochondria and ATP decrease, and in return aggravates the development of arrhythmia.

Following endothelial dysfunction and the buildup of LDL within the subendothelial region, macrophages uptake LDL through scavenger receptors, and acid lipase present in these cells catalyzes the hydrolysis of LDL into free cholesterol (12). In physiological states, cholesterol efflux in macrophages is activated by the LDL receptor—but not by scavenger receptors—via ABCA1 and ABCG1 transporters (13). Consequently, in atherosclerotic conditions, cholesterol progressively accumulates in macrophages, leading to their conversion into foam cells. The excessive deposition of cholesterol and additional lipids triggers ER stress and elicits inflammatory reactions within the plaque (14). More specifically, increased cholesterol levels in macrophages can promote the assembly of inflammasomes, the secretion of IL-1β, and the induction of NETosis (15). Moreover, 25-hydroxycholesterol has been found to induce the release of mitochondrial DNA and stimulate IL-1β secretion through AIM2 inflammasome activation (16). Collectively, these findings indicate that cholesterol metabolism modulates the inflammatory response of macrophages in the setting of atherosclerosis. In summary, disruptions in lipid metabolism within endothelial cells and macrophages constitute key mechanisms driving atherosclerosis (Fig. 1).

### Cardiomyopathy

Among all cardiomyopathy subtypes, modifications in lipid metabolism observed in diabetic cardiomyopathy have been investigated most comprehensively. Intramyocardial lipid overload in failing human hearts has also been demonstrated to be more pronounced in diabetic individuals compared to nondiabetic individuals (17). Elevated myocardial triglyceride accumulation has been linked to increased left ventricular mass as well as systolic and diastolic dysfunction (18, 19). This evidence suggests that lipid accumulation is the primary factor responsible for the initiation and progression of diabetic cardiomyopathy.

Mechanistically, the buildup of lipids can drive mitochondrial impairment; in turn, compromised mitochondrial activity may further result in the accumulation of lipotoxic medium-chain acylcarnitines, establishing a self-perpetuating cycle of dysfunction and concurrently enhancing reactive oxygen species (ROS) generation (20). These observations are corroborated by human studies, which show that myofibers isolated from the right atrial appendages of individuals with diabetes are characterized by lipid accumulation (21). Ultimately, excess FFAs can serve as ligands for PPAR-α, thereby inducing the upregulation of genes responsible for FFA uptake, transport, and oxidation (22). Cardiac-specific overexpression of PPAR-α leads to a phenotype that recapitulates diabetic cardiomyopathy, further aggravating lipid accumulation and perpetuating this detrimental cycle. The lipid-mediated activation of key pathological pathways—including inflammation, elevated ROS production, mitochondrial dysfunction, disrupted insulin signaling, apoptosis, and aberrant calcium handling—leads to downstream consequences such as contractile deficits, impaired cardiac relaxation, and fibrosis, ultimately culminating in cardiomyopathy. Supporting this concept, diabetic individuals display cardiac lipid accumulation concurrent with cardiac hypertrophy and impaired left ventricular systolic and diastolic function (18, 19). In young women with type 1 diabetes, myocardial fatty acid oxidation rates are elevated relative to nondiabetic controls (23). Likewise, patients with type 2 diabetes also demonstrate enhanced fatty acid oxidation (24). In summary, fatty acids are increased in diabetic cardiomyopathy, and lipid accumulation contributes to mitochondrial dysfunction (Fig. 1).

### Ischemic heart diseases

Lipid metabolism is critically involved in myocardial ischemia-reperfusion injury. During the ischemic phase, augmented fatty acid uptake and metabolic activity help sustain the equilibrium between myocardial energy requirements and available metabolic pathways. However, upon reperfusion, heightened fatty acid oxidation exacerbates oxidative stress in cardiomyocytes, thereby worsening myocardial ischemia-reperfusion injury (25). Recent investigations have uncovered that aberrations in cellular autophagy and mitochondrial function linked to lipid metabolism are central to ischemia-reperfusion injury (26, 27). Consequently, refining lipid metabolism and mitochondrial performance may offer an effective approach for preventing and managing myocardial ischemia-reperfusion injury. Examining lipid metabolism and its regulatory mechanisms provides a novel perspective for understanding the pathogenesis of this condition and offers robust support for the development of new therapeutic strategies.

Fatty acids typically serve as the principal substrate for myocardial oxidative metabolism, rendering efficient fatty acid oxidation especially vital during ischemia-reperfusion (28). Circulating fatty acid concentrations increase during and following ischemic episodes, resulting in accelerated fatty acid oxidation. Multiple studies have indicated that excessive fatty acid oxidation correlates with myocardial ischemic injury; accordingly, suppressing excessive fatty acid oxidation has been demonstrated to be protective against such damage. These results emphasize the significance of maintaining lipid metabolic homeostasis in the context of myocardial ischemia (Fig. 1).

### Cardiac arrhythmias

Given that atrial fibrillation (AF) represents the most prevalent cardiac arrhythmia, the associated alterations in lipid metabolism are of primary interest. During AF, the atrial rate ranges from 350 to 600 beats per minute, resulting in irregular and incomplete atrial contractions. These accelerated and arrhythmic contractions elevate energy demands and necessitate consecutive adaptive changes in metabolic supply. Apart from the increased rate, arrhythmic pacing has been associated with metabolic disturbances in murine cardiomyocytes in vitro (29). AF induces metabolic stress in both the atria and the ventricles (30). Moreover, AF accompanied by low cardiac output can trigger systemic metabolic stress, which has been demonstrated to be partially ameliorated by catheter ablation (31).

In patients with AF, a significant upregulation of ketone body metabolism has been observed in the atrial myocardium, marked by elevated activity of the enzyme t 3-oxoacid CoA transferase (SCOT), which is indispensable for ketone activation (32). Metabolomic analyses further substantiate this observation by revealing higher concentrations of β-hydroxybutyrate and specific ketogenic amino acids, such as tyrosine, whose catabolism yields acetoacetate and fumarate. Moreover, patients with AF exhibit an increased fumarate-to-succinate ratio. Interestingly, β-hydroxybutyrate generates more energy per carbon unit than pyruvate (33), a characteristic proposed to elicit a glucose-sparing effect in cardiac tissues. This effect occurs via the accumulation of acetyl CoA, which inhibits the pyruvate dehydrogenase complex (34). This complex serves as the link between glycolytic pyruvate and the tricarboxylic acid cycle; consequently, glucose is redirected toward glycogen synthesis. Indeed, in a rat model, introducing ketones during glucose perfusion triggered a metabolic shift from glycogenolysis to glycogenesis (35). This shift is paralleled in patients with AF, who display upregulated expression of glycogen synthase and downregulated expression of glycogen phosphorylase (36). Glycogen accumulation can lead to myolysis and alterations in cellular ultrastructure (37). Aberrant glycogen metabolism may exacerbate these cellular injuries, further promoting atrial fibrosis and cardiomyopathy. Furthermore, glycogen accumulation may affect the distribution and function of gap junction proteins (such as Cx43), thereby impairing intercellular electrical conduction in the myocardium (38). Collectively, these data indicate that upregulated ketone body metabolism in AF may inhibit glucose utilization and stimulate glycogen synthesis (Fig. 1).

### Heart failure

The centrality of lipid utilization for cardiac function is evidenced by obesity, a state characterized by heightened FFA levels (39). Upon exposure to a high-fat diet, the heart rapidly develops insulin resistance; however, the ablation of insulin signaling results in a relatively mild phenotype. Mice with a cardiac-specific GLUT1 knockout exhibit a 50% decrease in glucose uptake and utilization. Nevertheless, by enhancing fatty acid oxidation (FAO), these mice do not display a greater susceptibility to heart failure (HF) (40). GLUT1 transgenic mice also preserve normal cardiac function on standard chow diets, although they develop HF when maintained on high-fat diets (41). It has been suggested that reduced glucose uptake and increased fatty acid uptake into the heart are causative elements in diabetic cardiomyopathy and drive HF in the mouse models of diabetes (42).

Clinical observations provide further support: individuals exhibiting severe impairments in lipid oxidation manifest more compromised cardiac function. A deficiency in very long-chain acyl-CoA dehydrogenase disrupts mitochondrial lipid oxidation, resulting in altered muscle energy metabolism and ultimately cardiomyopathy (43). Intracellular lipid accumulation can arise from excessive mitochondrial lipid uptake, as documented in the mouse models of long-chain and very long-chain acyl-CoA dehydrogenase deficiency. While reduced FAO has been demonstrated in the failing heart by many studies, recent data indicate that in HF with preserved ejection fraction, cardiac FAO is elevated and glucose utilization is diminished, thereby enabling greater ROS generation (44) (Fig. 1).

## LDAPs and lipid metabolism

### Perilipins

Numerous investigations have confirmed that perilipin (PLIN) constitutes major LDAPs across diverse tissues and cell types, with certain studies explicitly establishing their essential role in LD generation. Initial research identified PLIN1 and PLIN2 in MA-10 cells and Y-1 adrenocortical cells (45), and Hsieh *et al.* demonstrated that treatment with oleic acid or cholesterol induced distinct PLIN proteins in mouse adrenal cortical cells and liver cells. During LD formation, PLIN4 and PLIN1c—a splice variant of PLIN1—were observed (46).

LDs containing PLIN are characteristically small particles, and an association with mitochondria in cardiac muscle cells was revealed via transmission electron microscopy (47). Specifically, PLIN were found in mitochondrial fractions isolated from cardiomyocytes. The establishment of PLIN-knockout (KO) mice by Kuramoto *et al.* demonstrated a central role for PLIN in mitochondrial energy production within muscle cells (48). In this study, even high magnification electron micrographs failed to detect LDs in cardiac muscles from PLIN-KO mice. However, some LDs were detected in soleus muscle myotubes after fasting. Although PLIN-KO mice appeared physically normal, they exhibited intolerance to endurance exercise. Furthermore, cultured cardiomyocytes from PLIN-KO mice displayed greater fatty acid oxidation than those from WT mice. Given that fatty acid β-oxidation is the primary energy source for cardiac muscles, these findings suggest that PLIN modulate fatty acid distribution and lipid metabolism by regulating LD formation (48).

### Cell death-inducing DNA fragmentation factor-like effectors

The cell death-inducing DNA fragmentation factor-like effector (CIDE) protein family, which includes CIDEa, CIDEb, and CIDEc, is characterized by its homology to the N-terminal domain of DNA fragmentation factor 40/45 (DFF40/45) (49). While initial research indicated that overexpressing these proteins in various cell types triggers caspase-independent apoptosis, later genetic evidence revealed a primary physiological role in lipid metabolism regulation (50). For example, investigations using CIDEa-deficient mice have demonstrated that CIDEa modulates lipid storage and degradation processes in brown adipocytes. Furthermore, studies utilizing either CIDE-KO mice or overexpression models suggest that CIDE proteins govern multiple lipid metabolic pathways, such as lipid storage and LD biogenesis (51). Collectively, these proteins are localized to the ER and LDs, where they play a critical role in maintaining lipid homeostasis.

The three CIDE members display distinct, tissue-specific patterns of expression and distribution. CIDEa is abundantly expressed in murine brown adipose tissue (BAT), while in white adipose tissue (WAT), the expression levels are relatively low. In humans, CIDEa is highly expressed in adipose tissue, encompassing both BAT and WAT. Additionally, CIDEa expression in humans is detectable in the liver, heart, and skeletal muscle, albeit at lower levels (52). Furthermore, expression occurs in the mammary, sebaceous, and meibomian glands. Given that CIDEa−/− animals are resistant to high-fat diet (HFD)-induced obesity, show reduced milk lipids, and have dry skin and hair, its expression strongly correlates with lipid storage and secretion in these tissues (53). For humans, CIDEb expression is primarily detected in the liver, with low expression levels in the intestine. In contrast, mouse CIDEb has only been reported to express in hepatocytes. Deficiency in CIDEb results in various defects in lipid metabolic pathways. WAT is the primary site for CIDEc expression, with moderate levels found in BAT. When fed a HFD or under obese conditions, CIDEc−/− mice show a dramatic reduction in fat storage capacity and exhibit a lipodystrophic and insulin-resistant phenotype. Comparable observations were reported in the WAT of a 14-year-old patient with a homozygous mutation in the CIDEc protein (CIDEC-E186X) (54). This patient displayed ectopic lipid accumulation in the liver, along with dyslipidemia, hypertension, and insulin-resistant diabetes. Overall, these findings demonstrate that by modulating LD formation, CIDE proteins are essential regulators of various aspects of lipid homeostasis in metabolic tissues.

### Abhydrolase domain containing 5

The functional importance of abhydrolase domain containing 5 (ABHD5) was initially identified due to its link with neutral lipid storage disease (NLSDI). To investigate the underlying biochemical defects in NLSDI, multiple research groups utilized cells obtained from affected patients. Initial biochemical analyses demonstrated that triacylglycerol (TAG) levels in skin fibroblasts from NLSDI patients were 20-fold higher than those in control cells from healthy individuals, even when cultured in lipid-depleted media. These studies further indicated that the excess lipid was stored in cytoplasmic LDs and that acid lipase activity in NLSDI cells remained unchanged, thereby ruling out a defect in lysosomal degradation pathways (55). Similarly, TAG accumulation in NLSDI fibroblasts could not be explained by alterations in fatty acid (FA) uptake, transport, or β-oxidation. Furthermore, no apparent changes were observed in the activities of glycerophospholipid biosynthetic enzymes or general lipases in NLSDI cells (56).

Defects in either lipid or cholesterol (57) metabolic pathways were also ruled out as the underlying basis for the substantial accumulation of TAG. However, research conducted by Williams *et al.* (56) identified a deficiency in TAG lipase activity, which impairs the degradation of TAG in NLSDI fibroblasts. This was further characterized as a specific impairment in the breakdown of long-chain FA-containing TAGs, leading to the conclusion that a long-chain TAG lipase is absent in these cells. In contrast, Coleman and colleagues (58) reported that TAG hydrolysis remains intact in NLSDI fibroblasts, proposing instead that TAG accumulation stems from accelerated TAG resynthesis. By employing pulse-chase experiments alongside the acyl-CoA synthetase inhibitor triacsin C, they attributed this phenomenon to a defect in the recycling of TAG-derived acylglycerols into phospholipids.

### Berardinelli-Seip congenital lipodystrophy type 2 (BSCL2)

Several investigations have established a connection between seipin function and the regulation of intracellular calcium (59). In Drosophila, a physical interaction was identified between Seipin and the ER calcium pump SERCA, indicating that Seipin acts as a positive regulator of SERCA activity. This interaction resulted in reduced ER calcium levels in seipin KO flies, and pharmacologically inhibiting ER-to-cytosol calcium efflux partially rescued the observed defects in fat body lipogenesis. A subsequent study confirmed that the depletion of ER calcium stores impaired mitochondrial calcium uptake, leading to concurrent deficiencies in the tricarboxylic acid cycle and overall mitochondrial function (60). These findings are supported by multiple later studies, which have consistently reported disruptions in calcium homeostasis across various seipin knockdown systems (61).

Recently, links between seipin, mitochondria, and calcium fluxes was established based on recent investigation utilizing human and mouse cells, as well as inducible seipin-depleted mice (62). These studies indicated that mitochondrial calcium uptake was impaired within hours of acute seipin depletion, whereas ER calcium stores were initially unaffected. This defect in mitochondrial calcium flux was associated with mitochondrial dysfunction in multiple cell types, including those derived from BSCL2 patients. Mechanistically, a portion of seipin was shown to localize to ER-mitochondria contact sites (MAMs) in a manner regulated by nutritional status, positioning itself near the calcium regulators SERCA and IP3R. These observations imply that seipin may be essential for MAM stabilization to enable mitochondrial calcium uptake.

A recent study corroborated the presence of seipin at MAMs in human cells (63). This research identified the Orp5/Orp8 complex as essential for LD formation, localizing to newly defined ER-MAM-LD tripartite contact sites proposed as the locations for LD emergence. Crucially, Orp5 was found to be necessary for recruiting seipin to these MAM regions, which are enriched in phosphatidic acid. Interestingly, the dynamic localization of seipin was observed to decrease at MAMs under lipogenic conditions and increase during starvation. Discrepancies between studies might be attributed to cell type-specific differences. Collectively, these findings suggest that cellular nutritional status modulates the MAM lipid environment, thereby driving seipin recruitment via Orp5; at these sites, seipin likely functions to stabilize calcium-regulating ER subdomains (64).

### Rab18

Rab18 is an ER-resident protein that facilitates the sustained interaction between nascent LDs and the ER, as described previously. Wilfling *et al.* utilized electron microscopy to visualize connections between the ER and LDs, which permit the transfer of enzymes involved in triglyceride synthesis, such as GPAT4 and DGAT2, from the ER to developing LDs, allowing for the in situ production of triglycerides on the LD surface. However, the precise molecular mechanisms underlying these connections remain unknown. Although Arf1 and COPI have been proposed by the same group to modify the phospholipid composition and surface tension of the LD surface to promote connections (65), there is currently no evidence to exclude classical Rab-regulated membrane tethering from being involved in this process. Rab18 could potentially coordinate various interactions between the ER and LDs, some of which may be primarily driven by ER-associated Rab18. Therefore, the observed absence of Rab18 on certain LDs (66) does not preclude a role for Rab18 in regulating the association of these LDs with the ER. It is also plausible that Rab18 remains associated with LDs throughout a portion of their life cycle.

Alternative investigations have suggested a function for Rab18 on the ER that is independent of LDs. According to Barr *et al.* (67), both Rab18 and Rab3GAP are necessary for maintaining normal ER architecture across various cell types. They observed that the absence of either protein, or the use of primary fibroblasts from Warburg-Micro patients, led to a suppression of tubular ER and a concomitant expansion of CLIMP-63-defined ER sheets toward the cell periphery. Based on these observations, they hypothesize that Rab18 contributes to the maintenance of normal ER structure, potentially indicating a distinct role in regulating reticulons or other proteins critical for ER morphology. This study did not exclude the possibility that inefficient transfer of fatty acids or triglycerides from the ER to LDs might result in toxic effects due to accumulation within the ER. In contrast, Jayson *et al.* claimed that deleting the Rab18 gene had no impact on ER morphology in a carcinoma cell line (68). Collectively, these findings reinforce the idea that Rab18 is a protein involved in the transfer of fatty acids or triglycerides from the ER to LDs.

## LDAPs in CVDs

### Atherosclerosis

**Fig. 2:**
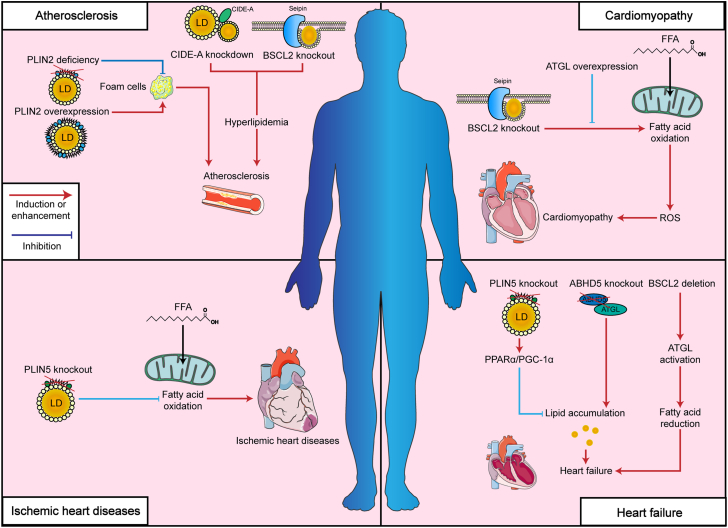
Schematic diagram of the roles of lipid droplets-associated proteins (LDAPs) in different CVDs. In atherosclerosis, PLIN2 overexpression promotes the formation of foam cells, while PLIN2 deficiency can inhibit lipid accumulation in the cells and prevent the disease. Moreover, CIDE-A knockdown and BSCL2 knockout can lead to hyperlipidemia and furtherly induce atherosclerosis. BSCL2 is also essential for myocardial fatty acid oxidation, and its knockout can accelerate ROS production and finally contribute to cardiomyopathy. In the ischemic heart diseases, PLIN5 knockout can inhibit the level of fatty acid oxidation and this interference can promote ischemic cardiac injury. Similarly, PLIN5 knockout can activate PPAR-α–PGC-1α pathway and reduce lipid accumulation. In contrast, ABDH5 knockout can increase lipid accumulation. Both decline and increase of lipid droplets can cause heart failure. In addition, BSCL2 deletion can activate ATGL and reduce fatty acid in cardiomyocytes and contribute to heart failure.

In the setting of atherosclerosis, the absence of PLIN2 in foam cells has been shown to decrease LD accumulation and confer protection against the development of atherosclerotic lesions (69). In contrast, elevated expression of PLIN2 promotes the buildup of triglycerides and cholesterol, thus enhancing LD formation (70). While there is still limited direct evidence connecting CIDE proteins to atherosclerosis, members of this gene family are strongly linked to hyperlipidemia, a key risk factor for the disease. For instance, mice lacking CIDEa exhibit markedly increased lipolysis and are protected from HFD-induced hyperlipidemia (71). Likewise, suppression of CIDEa in human adipocytes leads to enhanced fatty acid oxidation and a reduction in LD size (72). Moreover, systemic deletion of BSCL2, another gene implicated in lipolysis, in mouse models predisposed to atherosclerosis (Ldlr−/− or apoE−/−) results in severe hyperlipidemia, worsened steatohepatitis, and accelerated atherosclerotic progression (73). Together, PLINs, CIDEs, and BSCL2 play pivotal roles in regulating LD biogenesis and maintaining lipid homeostasis, thereby exerting significant influence on the development and progression of atherosclerosis (Table 1 and Fig. 2).

**Table 1:** Roles of LDAPs in lipid metabolism in CVDs

LDAPs		Animal models or clinical participants	CVDs	Influence of LDPAs on lipid metabolism	Influence of LDPAs on cardiovascular function	Reference
PLIN	PLIN5	PLIN5 knockout mice	-	Cultured cardiomyocytes from PLIN5-null mice had higher fatty acid oxidation level and more ROS	Mutant mice hearts had worse heart function	Kenta *et al.* (46)
	PLIN5	PLIN5 knockout mice	Ischemic heart disease	PLIN5 deficiency reduced lipid storage and elevated free fatty acids	PLIN5 deficiency exacerbated the myocardial infarct area, aggravated left ventricular systolic dysfunction	Zheng *et al.* (75)
	PLIN5	Cardiac-specific overexpression of PLIN5 in mice	Heart failure	PLIN5 overexpression mice showed reduced cardiac FA oxidation but normal ATP production	PLIN5 overexpression preserved of heart function	Stephanie *et al.* (95)
	PLIN5	PLIN5 knockout mice	Type 1 diabetes-induced heart malfunction	PLIN5 knockout mice lacked detectable LDs in the heart and did not exhibit aberrant lipid accumulation, excessive ROS generation	PLIN5 knockout had worse heart malfunction.	Kenta *et al.* (72)
	PLIN1	PLIN1 knockout mice	Hypertrophic cardiomyopathy	PLIN1 knockout had ectopic lipid accumulation and enhanced fatty acid transport and oxidation	PLIN1^−/−^ mouse hearts had injured myocardial structure and function	Liu *et al.* (77)
	PLIN2	Cardiac-specific overexpression of PLIN2	Atrial fibrillation	PLIN2-Tg mice showed accumulation of small lipid droplets around mitochondrial chains	PLIN2-induced steatosis is associated with Cx43 remodeling, impaired conduction propagation, and higher incidence of AF in aged mice	Satsuki *et al.* (96)
CIDEs	CIDEb	Liver-specific CIDEb-null mice	Atherosclerosis	Liver-specific CIDEb-null mice had lower rates of cholesterol biosynthesis	CIDEb-null mice had reduced cholesterol levels in the heart	Li *et al.* (50)
ABHD5	ABHD5	ABHD5 knockout and transgenic mice	Heart failure	ABHD5 deficiency led to neutral lipid storage disease in mice	ABHD5 expression protected from pressure overload-induced heart failure	Zegeye *et al.* (79)
BSCL2	BSCL2	Cardiomyocyte-specific deletion of BSCL2 mice	Cardiomyopathy	Myocardial BSCL2 deletion led to elevated ATGL expression and FAO along with reduced cardiac lipid contents	Mice with cardiac-specific deletion of BSCL2 developed systolic dysfunction with dilation	Zhou *et al.* (81)
	BSCL2	Seipin/Apoe double knockout mice	Hyperlipidemia	Seipin/Apoe double knockout led to significantly reduced hepatic steatosis and insulin resistance in mice	Seipin/Apoe double knockout led to significantly reduced hyperlipidemia	Meng *et al.* (84)
	BSCL2	Seipin/Apoe double knockout mice	Atherosclerosis	Seipin^−/−^ apoE^−/−^ mice also developed steatohepatitis and increased atherogenesis	Seipin−/−apoE−/− mice led to hyperlipidemia and atherosclerosis	*Liao et al.* (71)
DGAT1	DGAT1	DGAT1 knockout mice	-	Loss of DGAT1 activity in muscles decreases mRNA levels of genes involved in lipid uptake and oxidation	Loss of DGAT1 did not lead to heart failure	Liu *et al.* (97)
	DGAT1	Transgenic overexpression of DGAT1 mice	Lipotoxic cardiomyopathy	Transgenic overexpression of DGAT1 increased fatty acid oxidation	Induction of DGAT1 could be beneficial in the setting of excess heart accumulation of toxic lipids	Liu *et al.* (85)
	DGAT1	Cardiomyocyte-specific DGAT1 knockout mice	Heart failure	Cardiomyocyte-specific DGAT1 knockout mice. Hearts had 95% increased DAG and 85% increased ceramides	Cardiomyocyte-specific DGAT1 knockout mice had worse cardiac function	Liu *et al.* (86)
	DGAT1	Cardiac-specific overexpression of DGAT1 mice	Ischemic heart diseases	Cardiac-specific overexpression of DGAT1 elevated TAG turnover rates	Cardiac-specific overexpression of DGAT1 protected ischemia-reperfusion injury	*Stephen et al.* (98)
ATGL	ATGL	ATGL deficiency mice	Heart failure	ATGL deficiency in mice decreases mRNA levels of PPAR-α and PPAR-δ target genes, followed by excessive lipid accumulation	ATGL deficiency in mice cardiac insufficiency and lethal cardiomyopathy	Haemmerle G *et al.* (99)
	ATGL	Endothelium-specific deletion of ATGL mice	Atherosclerosis	Deletion of ATGL in the endothelium led to neutral lipid accumulation in vessels	Deletion of ATGL in the endothelium promoted atherosclerosis	Nabil *et al.* (11)
	ATGL	Adipocyte-specific ATGL knockout mice	Heart failure	ATGL inhibition reduced lipolysis in adipocyte	ATGL inhibition ameliorates isoproterenol-induced cardiac remodeling	Shingo *et al.* (100)
	ATGL	Tissue-specific deletion of ATGL mice	Heart failure	Phosphatidylethanolamines was attenuated in atATGL-KO hearts	atATGL-KO mice were protected against TAC-induced systolic LV failure	*Janek et al.* (101)
	ATGL	Cardiomyocyte-specific ATGL overexpression mice	Heart failure	ATGL overexpression reduced myocardial triacylglycerol content	ATGL overexpression protected against pressure overload-induced cardiac dysfunction	Petra *et al.* (102)
	ATGL	Cardiomyocyte-specific ATGL overexpression mice	Diabetic cardiomyopathy	ATGL increased in intramyocardial TAG levels, lipotoxicity	ATGL overexpression protected against lipotoxic cardiomyopathy	Thomas *et al.* (103)
	ATGL	Cardiomyocyte-specific ATGL overexpression mice	Dilated cardiomyopathy	Cardiomyocyte-specific ATGL overexpression induced decreased fatty acid uptake, storage, and oxidation	Cardiomyocyte-specific ATGL overexpression prevent cardiac steatosis and decrease fatty acid utilization	Pulinilkunnil *et al.* (103)

ABHD5, abhydrolase domain containing 5; ATGL, adipose triacylglyceride lipase; BSCL2, Berardinelli-Seip congenital lipodystrophy type 2; CIDEb, cell death-inducing DFF45 (DNA fragmentation factor 45)-like effector b; CVDs, cardiovascular diseases; DGAT1, diacylglycerol O-acyltransferase 1; LDAPs, lipid droplet-associated proteins; PLIN, perilipins; ROS, reactive oxygen species.

### Cardiomyopathy

In murine models of type 1 diabetic cardiomyopathy, elevated expression of PLIN5 promotes the buildup of triglycerides and lipotoxic intermediates, including diglycerides and ceramides, leading to the activation of PKC-related signaling pathways and increased ROS generation. Conversely, the lack of PLIN5 protects the heart by preventing lipid accumulation and maintaining normal cardiac function (74). Beyond its association with systemic hyperlipidemia, complete deficiency of BSCL2 leads to severe hypertrophic cardiomyopathy (75). Importantly, inhibition of ATGL has been shown to reduce excessive fatty acid oxidation and help preserve cardiac performance in BSCL2-deficient animals (76). Together, PLINs, CIDEs, and BSCL2 serve as essential modulators of LD dynamics, playing a central role in the development and progression of cardiomyopathy (Table 1 and Fig. 2).

### Ischemic heart diseases

Similar to its influence on cardiomyopathy, the knockout of PLIN5 was found to alleviate fatty acid oxidation and result in an expanded ischemic area following reperfusion (77). Supporting this preclinical evidence, individuals carrying the common noncoding polymorphism rs884164 exhibited diminished cardiac function (78) (Table 1 and Fig. 2).

### Heart failure

The deficiency in PLIN1 has been shown to trigger cardiac hypertrophy and fibrosis, ultimately contributing to the development of HF (79). Similarly, loss of PLIN5, another member of the PLIN family, leads to reduced lipid accumulation in cardiomyocytes along with upregulation of PPARα and PGC-1α, molecular changes that are implicated in the progression to HF (80). As previously highlighted, ABHD5 plays a critical role in lipolysis across multiple tissues. In the heart, ABHD5 functions as a serine protease capable of cleaving HDAC4, thereby influencing MEF2-mediated glucose metabolism. Furthermore, deficiency of ABHD5 results in excessive lipid buildup and promotes the onset of HF (81). Notably, chemical-induced disruption of ABHD5 expression in cardiomyocytes, such as by hemoglobin subunit beta, has been shown to worsen cardiac injury (82). With regard to HF, cardiomyocyte-specific deletion of BSCL2 leads to pronounced systolic and diastolic dysfunction, which may be explained by increased ATGL expression and diminished fatty acid levels (83) (Table 1 and Fig. 2).

## Therapeutic strategies targeting LDAPs

Research centered on LDs indicates that these specialized organelles are critically involved in the pathogenesis of CVDs. Given that LDAPs serve as key modulators of LD biology, they represent promising molecular targets for the development of novel therapeutic strategies against CVDs.

### Modulation of LDs biogenesis and accumulation

To meet the heart's substantial energy demands, a significant portion of fatty acids is imported from the plasma via the membrane receptor CD36 (84). However, the chronic intracellular accumulation of high concentrations of these fatty acids induces lipotoxicity, primarily mediated by diglycerides and ceramides. To mitigate this toxicity, the sarcoplasmic reticulum serves as the site for LD formation. Although LDs are essential for neutral lipid storage, abnormal LD size and quantity have been identified as common pathological features in various CVDs. Indeed, rapid LD biogenesis and excessive accumulation have been documented in the myocardium of both murine and human samples with CVDs. Therefore, modulation of LD biogenesis represents a potential therapeutic strategy for the prevention and treatment of CVDs.

As previously discussed, BSCL2 is a LDAP that plays a pivotal role in LD biogenesis. Genetic manipulation of BSCL2 has demonstrated therapeutic potential in treating hyperlipidemia in mouse models. The elevated triglyceride levels resulting from BSCL2 deficiency can be reversed by either seipin expression (85) or adipose tissue transplantation (86). Although no pharmacological agents currently target BSCL2 directly, its upstream regulator, diacylglycerol acyltransferase (DGAT1), has been extensively studied. DGAT1 encodes an enzyme that catalyzes the final step of triglyceride synthesis. Liu *et al.* reported that DGAT1 overexpression in the mouse heart leads to increased triglyceride accumulation and greater LD formation (87), whereas its deficiency results in severe heart failure (88). These findings underscore the beneficial role of DGAT1 in maintaining cardiovascular lipid homeostasis. Notably, DGAT1-targeted therapeutics have already been developed. In hyperlipidemic mouse models, inhibition of DGAT1 using acyl CoA or A-922500 effectively reduces plasma triglyceride levels (89). Moreover, the DGAT1 inhibitor pradigastat has shown significant antihyperlipidemic effects in clinical settings (90). Collectively, these data indicate that DGAT1, as an upstream regulator of BSCL2, modulates LD-related lipid metabolism, and pharmacological targeting of DGAT1 may offer promising strategies for the treatment of CVDs.

### Maintenance of lipolysis homeostasis

In contrast to the process of LD biogenesis, lipolysis refers to the catabolic hydrolysis of stored lipids, resulting in the release of fatty acids. These free fatty acids are subsequently transported to mitochondria to undergo oxidative phosphorylation. Additionally, they can be chaperoned out of the adipocyte, for example by the FA binding protein aP2/FABP4, for uptake by other tissues, such as skeletal muscle or cardiac muscle (91, 92). In pathological conditions such as myocardial ischemia-reperfusion injury, a surge in intracellular fatty acid levels occurs, a phenomenon driven in part by excessive lipolysis. Therefore, understanding the mechanisms underlying lipolysis is of equal importance to studying lipid biogenesis for deciphering metabolic dysregulation in CVDs.

ATGL acts as the rate-limiting enzyme that catalyzes the initial stage of TAG hydrolysis. Originally identified in adipose tissue, its activity is enhanced by the co-activator CGI-58 (also known as ABHD5) (93). Mice with global genetic ablation of ATGL exhibit pronounced systemic lipid accumulation (94). PLIN5, a LDAP essential for regulating both LD biogenesis and turnover, physically interacts with ATGL and CGI-58 to tightly control lipolytic rates (95). Mechanistically, PLIN5 serves as a physiological inhibitor of ATGL by blocking the interaction between ATGL and its co-activator CGI-58 (96). Collectively, the interplay between the lipase ATGL and the coat protein PLIN5 is fundamental for maintaining cardiac LD homeostasis.

Therefore, the pharmacological inhibition of excessive lipolysis by targeting ATGL emerges as a promising therapeutic approach for CVDs, particularly ischemic cardiomyopathy. In pursuit of this goal, Atglistatin has been developed as a highly specific ATGL inhibitor. In models of isoproterenol-induced myocardial injury, treatment with Atglistatin conferred cardioprotection by reducing cardiomyocyte apoptosis and fibrosis, pathological processes driven by excessive fatty acid flux from adipose tissue lipolysis (97). Likewise, Atglistatin demonstrated significant therapeutic efficacy in murine models of heart failure, highlighting its potential for clinical application (98). In summary, modulating ATGL-mediated lipolysis—either through endogenous regulators such as PLINs or pharmacological agents like Atglistatin—represents a novel and promising avenue for the treatment of CVDs.

## Conclusion and Perspectives

Over the last several decades, extensive investigations have been conducted to characterize lipid metabolism under both physiological and pathological states. Concomitantly, efforts have been directed toward elucidating the molecular mechanisms that govern this metabolic process, with the goal of identifying novel therapeutic targets for CVDs. It is now well recognized that dysregulated lipid metabolism is a defining feature of various CVDs. When lipid oxidation is compromised or excessive lipid biogenesis takes place in cells such as cardiomyocytes and endothelial cells, surplus lipid metabolites—triglycerides in particular—are stored in specialized organelles known as LDs. Despite these advances, the precise mechanisms underlying LD regulation remain incompletely understood. Owing to progress in molecular biology, several critical LDAPs—including the PLIN family, BSCL2, CIDEs, ABHD5, and Rab18—have been discovered. These proteins maintain LD homeostasis by controlling either LD biogenesis or lipolysis, and genetic manipulation of these LDAPs has been shown to markedly influence the pathogenesis of CVDs. The functional importance of LDAPs underscores their potential as targets for therapeutic development and highlights the need for further studies to identify additional LDAPs involved in cardiovascular lipid metabolism. Such research endeavors hold significant promise for improving the diagnosis and treatment of CVDs. Although challenges remain in translating these findings to the clinic, therapies directed at LDAPs represent a highly promising frontier.

In summary, LDAPs are critically involved in the pathogenesis of CVDs through their regulation of lipid metabolic imbalances, and continued research into these proteins is expected to uncover novel molecular targets that may be leveraged for the treatment of these disorders (99).

## Data availability

All data presented in this paper can be found in the articles listed in the reference section.

## Conflicts of interest

The authors declare that they have no conflicts of interest with the contents of this article.
